# Plastic Materials in the Preparatory Treatment of Teeth

**Published:** 1891-05

**Authors:** Jacob L. Williams


### PLASTIC MATERIALS IN THE PREPARATORY TREATMENT OF TEETH.[1]

BY JACOB L. WILLIAMS, M.D.

In the whole scope of the practice of oristry perhaps there is no subject that more constantly presents itself than the need of avoidance of pain, together with the preservation of vitality in the organs to which the larger part of our attention is given.

Preparatory treatment of living teeth, of which only I shall speak, may be advisable in two classes of cases,—one where the pulp is endangered but not exposed, for the purpose of pulp protection, by arrest of caries and favoring deposit of secondary dentine; and in another class, where the dentine is too sensitive

---

[1] Read before the American Academy of Dental Science, Boston, January 7, 1891.

to permit without discomfort the proper preparation of cavities for permanent filling.

I will briefly mention how I first came to devise a plan for the treatment of the former class of cases, and which resulted in an adaptation for the treatment of the second class.

About the year 1850, and during my early practice with Dr. Keep, many useful teeth that were too frail to bear hard metallic fillings, instead of being doomed to extraction, were filled in various extemporized ways, to render them comfortable while they might last.

A material called Hill's stopping, coming forward about that time, was somewhat used, but, being a mixture of sulphate of lime with gutta-percha, it would become rough or fuzzy in the mouth; also being patented was considered an obstacle to its free use. So oxide of tin with a little fine spar was mixed with gutta-percha, which made quite a solid and comparatively durable stopping. But it had a serious objection in that it became discolored and often discolored the tooth substance.

Thinking over the matter frequently, it occurred to me one day that oxide of zinc might be free from these objections, and on trial I found it to be the fact.[1]

As it was common then to take pride in exclusive knowledge, I kept the secret to myself for some time, only loaning it to two students, one of whom gave it the name of "dentron." They derived some income by making and selling it to Codman & Shurtleff, who found a ready sale for it. When those students came to have no further occasion for that resource, I told the before-named firm of its composition, and they then began to make it for themselves. And being then freely spoken of, knowledge of the composition gradually became more general, and other dealers made it.

To resume. While using the tin combination I noticed, on removing the old stoppings for renewal, that the dentine had become *hardened* as well as discolored, and pulps that were before almost exposed were found protected by a firm covering of secondary dentine; and the thought came to me, Why could not a plan of treatment be adopted for the purpose of favoring this corrective and conservative action?

I reasoned that the morbid conditions in the cavity must first be corrected, and then held in suppression to allow nature to carry

[1] I cannot find any evidence that this combination had ever been made before.

out her protective efforts. Those morbific elements seemed to be fermentive and acid action. We knew nothing of microbes then, and what was the acid I knew not, but Dr. Miller has since informed us that it is mainly lactic.

To correct the fermentative process I saturated the cavity, after slight excavations, with a solution of chloride calcis, and to neutralize directly the acid. I used simple aqua calcis, each of which produced the desired effect.

But then the point was, how to place and hold the mischievous agents in subjection without irritating or destroying the subjacent vital tissues on which depended the renewed protection.

To accomplish this there then seemed to be nothing better at hand than creosote; but that used pure would be too caustic, so I diluted it *largely*, and saturated the cavity with the weak solution, and sealed it up; but how? I reasoned that the interior of a tooth, even in health, being softer and more elastic than other parts of it, something of a similar lack of density, as well as non-conductivity, should occupy the depth of the cavity.

Even the gutta-percha stopping would be too solid for some cases; and for them I mixed beeswax with it for the deeper layer, covering it with the firmer material. I also found that a bedding of oxide of zinc or of sulphate of lime mixed with the mild antiseptic answered the purpose admirably.

These corrective applications were repeated at intervals varying with the apparent needs of the cases.[1]

I made a brief mention of this plan in the *American Journal of Dental Science* in 1858.

In the course of time other antiseptics have been added to our armamentarium, giving greater variety of facilities in this line.

The principle of this plan of treatment that I have described is to destroy the morbific agents in the cavity, and keep it aseptic without destroying the living tissues, or so irritating them as to endanger destructive inflammation. And during all the years of practice on this principle, patiently followed, I have found its results successful, even beyond my early expectation. It sometimes seems to require almost an unreasonable amount of patience, but we must learn to wait on nature, or she will not help us.

In regard to corrective applications, how often are caustics or irritants used, perhaps with the idea that the strength of the cor-

---

[1] This new plan of treatment of course met with many amusing attempts at criticism.

rective will insure the duration of its effect, without considering that it may, as it often does, destroy the desired vital protective action!

And in relation to the relative density of interior dentine to the rest of the tooth, how often do we find an endangered pulp struggling in its minute pulsations against an unyielding phosphate stopping that lies almost or quite in contact with it!

And still further, how often have we seen a hard filling of malleted gold or of analgam, in a deep cavity, supposed to be safe, but by its relentless presence provoking rebellious inflammation and death of the pulp!

In following up the treatment for endangered pulps, it was readily observed that the marginal dentine also was obtunded, and often only by the presence of the plastic stopping, though the calcic saturation seemed to be a positive aid.

And this manner of obtunding extremely sensitive dentine in preparation for permanent filling I cannot but think preferable to the way of merely putting the fibrils to sleep, from which to wake and find themselves imposed upon by a rigid and temperature-conducting foreign substance, provoking them to neuralgic protests.

